# Transfer of Caffeine and Its Major Metabolites to Chicken Eggs

**DOI:** 10.3390/ani14131856

**Published:** 2024-06-22

**Authors:** Mailson da Silva Teixeira, Carolina Julia Costa Saraiva, Bárbara Pereira dos Santos, Thais Cristina Vann, Leonardo José Camargos Lara, Benito Soto-Blanco

**Affiliations:** 1 Laboratório de Toxicologia Veterinária, Departamento de Clínica e Cirurgia Veterinárias, Escola de Veterinária, Universidade Federal de Minas Gerais, Belo Horizonte 31275-013, Brazil; mailson.medvet@gmail.com (M.d.S.T.); carolinatoxicologia@gmail.com (C.J.C.S.); barbaraps.1711@gmail.com (B.P.d.S.); 2 Programa de Pós-Graduação em Biociências, Universidade Federal de Ciências da Saúde de Porto Alegre, Porto Alegre 90050-170, Brazil; thaisvann@hotmail.com; 3 Departamento de Zootecnia, Escola de Veterinária, Universidade Federal de Minas Gerais, Belo Horizonte 31275-013, Brazil; leolara@vet.ufmg.br

**Keywords:** theobromine, theophylline, paraxanthine, laying hens, residues in food

## Abstract

**Simple Summary:**

Caffeine is a natural substance found in coffee (*Coffea* spp.), cocoa (*Theobroma cacao*), cola (*Cola nitida*), guaraná (*Paullinia cupana*), maté (*Ilex paraguariensis*), and tea plant (*Camellia sinensis*). These plant species are widely used to produce different drinks and foods, generating a large volume of by-products that may be used for animal feed. This article is part of a broad study that aims to evaluate the safety of using by-products containing caffeine to feed laying hens. In this study, we show that the eggs of laying hens fed caffeine contain this compound together with its primary metabolites: theophylline, theobromine, and paraxanthine. It is essential to highlight that the levels of these compounds found in eggs do not appear to pose any health risk to consumers.

**Abstract:**

This study aimed to determine whether the eggs of laying hens fed caffeine contain this compound and its primary metabolites (theophylline, theobromine, and paraxanthine). Laying hens were distributed into four experimental groups fed rations containing 0 (control), 150, 300, or 450 μg/g of caffeine. For residual analysis, six eggs per group were collected after 4, 8, and 12 weeks. The concentrations of caffeine, theophylline, theobromine, and paraxanthine were determined in the white and yolk of each egg by a high-performance liquid chromatography with photodiode array detector (HPLC-PDA) method. All four compounds were detected in the white and yolk of eggs produced by hens fed caffeine, but their levels in the egg white were approximately twice those in the yolk. The major metabolite found in eggs was theophylline (57.5% of caffeine metabolites in the egg white and 58.5% in the yolk), followed by theobromine (39.9% in the egg white and 41.5% in the yolk), and paraxanthine (2.64% in the egg white and non-detected in the yolk). In summary, caffeine and its metabolites, theophylline, theobromine, and paraxanthine, are transferred to the chicken eggs.

## 1. Introduction

Purine alkaloid caffeine (1,3,7-trimethylxanthine) is a well-known adenosine A1, A2A, and A2B receptor antagonist in the central nervous system and peripheral tissues [1]. The maximum daily caffeine intake considered safe by the European Food Safety Authority is 200 mg [2]. Although there is vast individual variability in caffeine tolerance, excessive consumption can interfere with the cardiovascular, central nervous, reproductive, and gastrointestinal systems, calcium metabolism, liver, kidneys, and muscles [3]. The main biotransformation of caffeine is catalyzed by the CYP1A2 isoform of cytochrome P450, generating metabolite paraxanthine (about 84%), theobromine (about 12%), and theophylline (about 4%) [4]. All these metabolites are known to have active pharmacological activities and variable toxicity, with theophylline being the highest and paraxanthine the lowest [3].

The best-known plant containing caffeine is coffee (*Coffea* spp.), but this substance is also found in cocoa (*Theobroma cacao*), cola (*Cola nitida*), guaraná (*Paullinia cupana*), maté (*Ilex paraguariensis*), and tea plant (*Camellia sinensis*) [5]. These plant species are widely used to produce different drinks and foods, generating a large volume of by-products that may be used for animal feed [6,7]. However, the inclusion of coffee husks [6], cocoa bean shells [8], and green tea powder [9,10] as fiber sources in the diet of laying hens may negatively impact egg production and quality parameters, which is caused by caffeine [11]. Using by-products containing caffeine for laying hens feed is safe when the diet levels of this xanthine are less than 150 μg/g, as no significant harmful effects on egg production and quality were observed at this level [11]. As the average contents of caffeine in coffee husks, cocoa bean shells, and green tea are 10.0 mg/g, 4.21 mg/g, and 25.9 mg/g, the diets for laying hens should not contain more than 15 kg/ton of coffee husk, 35.6 kg/ton of cocoa bean shell, or 5.7 kg/ton of green tea powder [11].

A limitation to using caffeine-containing ingredients in feeding laying hens is that it is not known whether caffeine or its metabolites can be transferred to eggs. Thus, this study aimed to determine whether the eggs of laying hens fed caffeine contain this compound and its primary metabolites (theophylline, theobromine, and paraxanthine).

## 2. Materials and Methods

### 2.1. Animals and Treatment

The study’s experimental design was approved by the Animal Use Ethics Committee of UFMG (protocol #28/2018). The present study used eggs produced by 576 Lohmann LSL^®^ laying hens aged 56 weeks at the start of the experiment; all hens were added to the experiment on the same day. These animals were the same as those from an earlier published study [11]. Hens were housed in cages of 2000 cm^2^, at a density of 6 hens per cage. A wood splitter was used to isolate animal partitions and prevent the access of hens to the feed from another partition. The lighting of the laying house was 14 h of light/day, composed of 12 h of natural light, one hour of artificial light at dawn, and another hour of artificial light in the early evening. Water and food were provided ad libitum.

Laying hens were distributed into four experimental groups and were fed rations containing 0 (control), 150, 300, or 450 μg/g of caffeine. Each group was formed by 144 hens, distributed in 24 replicates of 6 hens. The choice of caffeine concentrations in the diet was based on a study that identified that the inclusion of 42.5 g/kg of coffee husks in the diet of laying hens worsened egg production [6]. We estimated that this amount of coffee husk would be equivalent to 425 μg of caffeine per g of feed [11].

The composition of the basal diet is presented in Table 1. Caffeine (anhydrous caffeine, Sulfal, Belo Horizonte, MG, Brazil) was added to the ration during its production at the feed factory to ensure adequate mixing of this compound in the ration. For residual caffeine and metabolites analysis, six eggs per group were collected after 4, 8, and 12 weeks.

### 2.2. Analytical Method

The concentrations of caffeine, theophylline, theobromine, and paraxanthine were determined in the white and yolk of each egg by a high-performance liquid chromatography with photodiode array detector (HPLC-PDA) method.

The measurement of caffeine in eggs was based on the method described by Summa et al. [12]. A sample of 5 g of the egg white or yolk was put in a 15 mL polypropylene tube, mixed with 0.5 mL of 2% acetic acid and, after 15 min, 5 mL of chloroform was added. The mixture was homogenized in a vortex for one minute and centrifuged at 5000 rpm for 20 min. Magnesium sulfate (4 g) and sodium chloride (2 g) were added, and the tubes were vortexed, centrifuged, and maintained in a freezer (−20 °C) for two hours. After that, the chloroform fraction was separated into another tube and dried. The dried extract was solubilized in 10 mL of deionized water, and the solution was put on a solid phase extraction (SPE) C8 cartridge (CHROMABOND C8, 45 µm, 6 mL/500 mg, Macherey-Nagel, Düren, Germany) previously conditioned with 5 mL of water, 5 mL of methanol, and 5 mL of water. Caffeine was eluted with 2 × 0.5 mL of methanol:2% acetic acid in a water solution (70:30, *v*:*v*). The eluate was injected directly into the HPLC.

Chromatographic analyses were performed in an HPLC system (Shimadzu Prominence LC-20A, Kyoto, Japan) equipped with a diode array detector (SPD-M20A, Shimadzu, Kyoto, Japan). Chromatographic separations were carried out on a Welch Welchrom C18 column (4.6 × 150 mm, 5 μm). The injection volume was 20 μL, and the mobile phase was methanol–water (25:75) at an isocratic flow of 1.0 mL/min. The detection wavelength was 274 nm, and the spectra were recorded from 190 to 400 nm. The total chromatographic run time was 12 min.

### 2.3. Method Validation

Validation was performed following the European Union SANTE/12682/2019 guidelines [13] using blank whole eggs. The following analytical performance parameters were assessed: linearity, selectivity, trueness, precision (repeatability and within-lab-reproducibility), limit of detection (LOD), and limit of quantification (LOQ). Standard calibration curves (0 to 500 μg/mL) were obtained using pure caffeine, theophylline, theobromine, and paraxanthine.

The trueness and precision (repeatability and within-lab-reproducibility) were determined from the recovery assay results of blank eggs spiked with caffeine, theophylline, theobromine, and paraxanthine at two distinct levels (5 and 25 µg/g). Repeatability was evaluated using data from replicate samples (*n* = 6) analyzed on the same day for each level. The within-lab reproducibility was evaluated using replicate data from three different days (*n* = 18). Repeatability and within-lab-reproducibility are expressed by the relative standard deviation (RSD in %), whereas average recovery values express trueness. The expanded measurement uncertainty (U) was estimated using the top-down approach. Precision deviations of up to 20% were considered acceptable [13].

The LOQ was determined to be the lowest concentration level of the calibration curve with acceptable accuracy. The LOD corresponded to 50% of the estimated value for the quantification limit, provided that the recoveries presented an area greater than or equal to 50% of the point in the matrix solution injected and the signal/noise ratio was higher than or equal to 3.

### 2.4. Statistical Analysis

The results are presented as mean ± standard deviation (SD). Data were analyzed using the Kruskal–Wallis test followed by Student–Newman–Keuls. The level of statistical significance was set at *p* < 0.05.

## 3. Results

### 3.1. Method Validation

Table 2 presents the HPLC method’s average recovery, repeatability, and expanded measurement uncertainty. For all compounds, the limit of detection was 0.15 µg/mL, and the limit of quantification was 0.50 µg/mL.

### 3.2. Analyses of the Eggs

**Figure 1:**
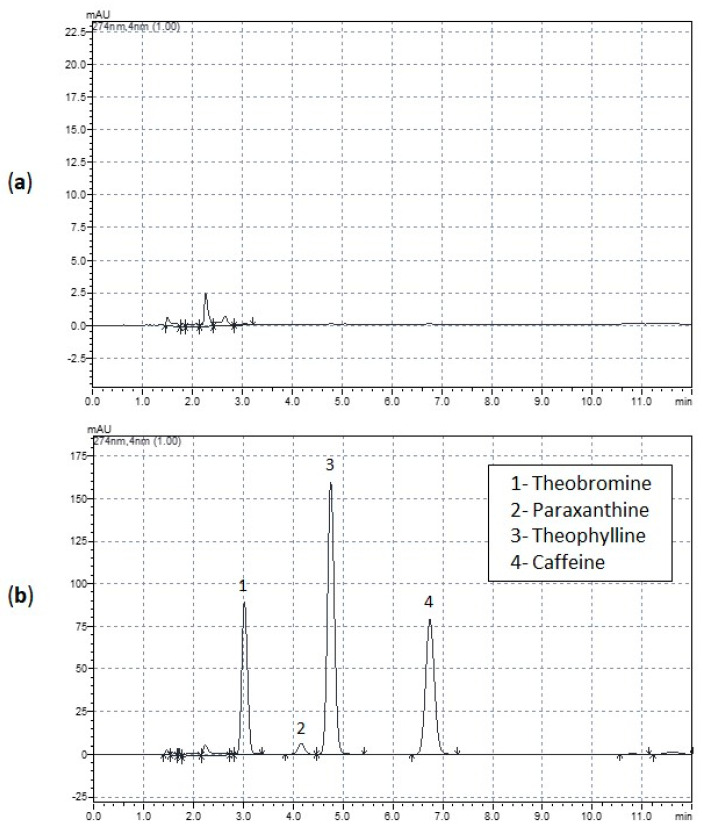
Chromatograms of egg whites. (**a**) Chromatogram of an egg white from the control laying hen fed a diet without caffeine for 12 weeks; (**b**) chromatogram of an egg white from a laying hen fed diet containing 450 μg/g of caffeine for 12 weeks showing the presence of theobromine, paraxanthine, theophylline, and caffeine.

**Figure 2:**
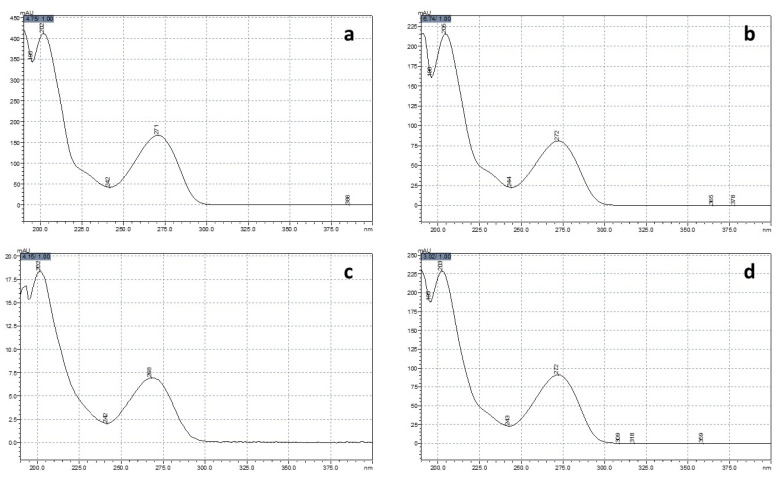
UV-visible spectra of theobromine (**a**), paraxanthine (**b**), theophylline (**c**), and caffeine (**d**) found in an egg white from a laying hen fed a diet containing 450 μg/g of caffeine for 12 weeks.

Caffeine, theophylline, theobromine, and paraxanthine were found in the egg whites of laying hens that consumed caffeine, but none of them was detected in eggs from the control group (Figure 1 and Figure 2, Table 3). All four compounds’ levels were statistically higher in egg whites from hens fed 300 μg/g and 450 μg/g than in control hens.

In the yolk, caffeine, theophylline, and theobromine were also found (Table 4), but not paraxanthine. All three compounds were found to be statistically higher in yolks from hens fed 300 μg/g and 450 μg/g than in controls.

## 4. Discussion

In the present study, it was found that eggs from laying hens that consumed caffeine showed residues of this substance and its metabolites, theophylline, theobromine, and paraxanthine. The concentrations of these compounds found in the egg white were approximately twice those in the yolk.

In humans, the primary caffeine metabolites are paraxanthine (about 84%), theobromine (about 12%), and theophylline (about 4%) [4]. In the present study, the major metabolite found in eggs was theophylline (57.5% of caffeine metabolites in the egg white and 58.5% in the yolk), followed by theobromine (39.9% in the egg white and 41.5% in the yolk), and paraxanthine (2.64% in the egg white and non-detected in the yolk). This result indicates that caffeine metabolism in chickens differs from that in humans. In fact, differences in this metabolism are known to occur in other animal species due to variations in cytochrome P450 (CYP) enzymes [14,15,16].

The present study is part of a large project aiming to determine the effects of caffeine consumption by laying hens to establish the amounts of caffeine-containing ingredients in their diets that do not cause negative consequences. As the first part of the study, the egg production and quality were published earlier [11]. The consumption of diets containing 300 and 450 μg/g caused a reduction in feed consumption, egg production, and eggshell thickness and percentage and an increase in the egg yolk percentage. Furthermore, hens fed ration containing 450 μg/g for 12 weeks showed a higher mortality rate than controls (1.45% vs. 0.23% per week) [11].

Residual xenobiotics in food must be evaluated for consumers’ safety. The European Food Safety Authority has established that the maximum safe dose of caffeine is 200 mg per day [2]. Theophylline appears to have a slightly higher toxicity than caffeine, while theobromine is less toxic, and paraxanthine has very low toxicity [3]. Considering an average weight of 66 g per egg, the average caffeine content found in the eggs of the hens that received the highest dose was 0.75 mg, and the sum of caffeine and metabolites was 3.2 mg. Therefore, consuming eggs from chickens fed with products containing caffeine should not pose any health risk.

Caffeine in fertilized eggs may impair embryo development [17,18,19,20,21,22]. Caffeine at concentrations from 2.5 μmol/egg (485.5 µg/egg) impaired chicken embryo development and increased embryo mortality and abnormality rates [17,21]. Changes found in chicken embryos incubated with caffeine include imperfect development of the thorax [21], abdomen, and organs [17]; several interferences in nervous system formation, including defective neural tube closures [17,18]; and effects on eye development including reduced retinal thickness [19,22] and asymmetrical microphthalmia [19]. Furthermore, caffeine inhibited angiogenesis in the yolk sac and chorioallantoic membranes [20]. However, the maximum concentration of caffeine in eggs that could not impair the development of the chick embryo is unknown.

## 5. Conclusions

Caffeine and its metabolites are transferred to the chicken eggs. The major metabolite found in eggs was theophylline (57.5% of caffeine metabolites in the egg white and 58.5% in the yolk), followed by theobromine (39.9% in the egg white and 41.5% in the yolk), and paraxanthine (2.64% in the egg white and non-detected in the yolk). The concentrations of these compounds found in the egg white were approximately twice those in the yolk.

## Figures and Tables

**Table 1:** Composition and nutritional values of the basal diet.

Ingredients	Amount (%)
Corn grain	62.0
Soybean bran (45% CP ^1^)	20.402
Wheat bran	3.00
Calcitic limestone	9.82
Meat and bone meal (40% CP)	4.00
Salt	0.38
Vitamin and mineral supplement ^2^	0.20
DL-methionine	0.14
L-Lysine	0.04
**Nutritional values**	**Amount**
AMEn ^3^ (kcal/kg)	2678.0
Crude protein (%)	16.4
Available phosphorus (%)	0.32
Calcium (%)	4.05
Sodium (%)	0.182
Digestible lysine (%)	0.747
Digestible Met + Cys (%)	0.588
Digestible methionine (%)	0.368
Digestible threonine (%)	0.540

^1^ CP—crude protein. ^2^ Composition per kg of product: Vitamin A: 8,000,000 UI, Vitamin D3: 2,100,000 UI, Vitamin E: 7000 mg, Vitamin K3: 2000 mg, Vitamin B1: 1000 mg, Vitamin B2: 3000 mg, Vitamin B6: 700 mg, Vitamin B12: 6000 mg, Folic acid: 100 mg, Biotin: 10 mg, Niacin: 20 g, Pantothenic acid: 2000 mg, Manganese: 55,000 mg, Zinc: 40,000 mg, Iron: 50,000 mg, Cupper: 6000 mg, Cobalt: 100 mg, Iodine: 1000 mg, Selenium: 200 mg, and Calcium: 10,000 mg. ^3^ AMEn—Nitrogen-corrected apparent metabolizable energy.

**Table 2:** Recovery (in %), repeatability relative standard deviation (RSD; in %), and expanded measurement uncertainty (U; in %) of the analytical method using blank eggs spiked with caffeine, theophylline, theobromine, and paraxanthine at 5 and 25 µg/g concentrations.

Compound	Recovery (%)	RSD (%)	U (%)
Caffeine			
5 µg/g	77.5	0.41	2.37
25 µg/g	83.5	0.30	4.60
Theophylline			
5 µg/g	66.9	0.54	1.94
25 µg/g	68.0	0.23	3.14
Theobromine			
5 µg/g	70.1	3.25	3.36
25 µg/g	84.1	0.35	2.00
Paraxanthine			
5 µg/g	63.5	0.98	2.09
25 µg/g	72.0	0.23	2.77

**Table 3:** Concentrations (in µg/g) of caffeine, theophylline, theobromine, and paraxanthine in the egg whites from laying hens fed diets containing different levels of caffeine.

Caffeine in Diet	Compound	Experimental Period		
		4 Weeks	8 Weeks	12 Weeks
0 (control)	Caffeine	0 ^a^	0 ^a^	0 ^a^
	Theophylline	0 ^a^	0 ^a^	0 ^a^
	Theobromine	0 ^a^	0 ^a^	0 ^a^
	Paraxanthine	0 ^a^	0 ^a^	0 ^a^
150 μg/g	Caffeine	3.84 ± 2.20 ^b^	1.18 ± 0.91 ^a,b^	1.51 ± 1.26 ^a^
	Theophylline	6.83 ± 1.24 ^a,b^	3.66 ± 2.46 ^a,b^	4.63 ± 3.76 ^a,b^
	Theobromine	4.93 ± 0.88 ^a,b^	2.58 ± 1.55 ^a,b^	3.20 ± 1.52 ^a,b^
	Paraxanthine	0.29 ± 0.06 ^a,b^	0.14 ± 0.08 ^a,b^	0.21 ± 0.19 ^a,b^
300 μg/g	Caffeine	6.05 ± 3.39 ^b^	3.97 ± 1.97 ^b,c^	3.17 ± 1.94 ^b^
	Theophylline	11.52 ± 4.38 ^b,c^	8.74 ± 3.55 ^b,c^	8.41 ± 3.97 ^b,c^
	Theobromine	7.52 ± 3.18 ^b,c^	5.43 ± 2.47 ^b,c^	5.67 ± 2.40 ^b,c^
	Paraxanthine	0.57 ± 0.24 ^b,c^	0.31 ± 0.17 ^b,c^	0.37 ± 0.15 ^b,c^
450 μg/g	Caffeine	5.18 ± 4.00 ^b^	11.47 ± 5.16 ^c^	3.83 ± 2.19 ^b^
	Theophylline	17.75 ± 5.52 ^c^	15.35 ± 6.22 ^c^	12.88 ± 4.28 ^c^
	Theobromine	12.62 ± 3.09 ^c^	12.01 ± 2.51 ^c^	8.84 ± 2.12 ^c^
	Paraxanthine	1.10 ± 0.42 ^c^	0.72 ± 0.27 ^c^	0.59 ± 0.18 ^c^

Data are shown as mean ± SD. ^a,b,c^ Different letters for each compound in the same column show significant differences (*p* < 0.05, Kruskal–Wallis test followed by Student–Newman–Keuls test). *n* = 6.

**Table 4:** Concentrations (in µg/g) of caffeine, theophylline, theobromine, and paraxanthine in the yolk of eggs from laying hens fed diets containing different levels of caffeine.

Caffeine in Diet	Compound	Experimental Period		
		4 Weeks	8 Weeks	12 Weeks
0 (control)	Caffeine	0 ^a^	0 ^a^	0 ^a^
	Theophylline	0 ^a^	0 ^a^	0 ^a^
	Theobromine	0 ^a^	0 ^a^	0 ^a^
150 μg/g	Caffeine	1.90 ± 0.85 ^a,b^	1.72 ± 0.84 ^b^	1.34 ± 1.20 ^a,b^
	Theophylline	2.57 ± 0.73 ^a,b^	2.36 ± 1.10 ^a,b^	2.57 ± 1.97 ^a,b^
	Theobromine	1.64 ± 0.72 ^a,b^	1.63 ± 0.66 ^a,b^	1.96 ± 1.35 ^a,b^
300 μg/g	Caffeine	3.55 ± 1.62 ^b,c^	2.38 ± 2.12 ^b,c^	2.95 ± 2.01 ^b,c^
	Theophylline	3.49 ± 1.37 ^b,c^	3.56 ± 2.63 ^b,c^	5.12 ± 2.54 ^b^
	Theobromine	2.31 ± 0.94 ^b,c^	2.79 ± 1.71 ^b,c^	3.82 ± 1.44 ^b^
450 μg/g	Caffeine	4.53 ± 1.40 ^c^	5.01 ± 1.11 ^c^	4.06 ± 0.99 ^c^
	Theophylline	4.99 ± 1.10 ^c^	7.06 ± 2.42 ^c^	5.89 ± 1.43 ^b^
	Theobromine	3.47 ± 0.81 ^c^	5.08 ± 0.91 ^c^	3.91 ± 1.06 ^b^

Data are shown as mean ± SD. ^a,b,c^ Different letters for each compound in the same column show significant differences (*p* < 0.05, Kruskal–Wallis test followed by Student–Newman–Keuls test). *n* = 6.

## Data Availability

The datasets generated for this study are available upon request from the corresponding author.
